# Late Breaking: Community-Engaged Research Approaches I Coping with Late Life Loss and Bereavement: The Healing Power of Transcendence and Creativity

**DOI:** 10.1093/geroni/igaf122.4348

**Published:** 2025-12-31

**Authors:** Jasmin Tahmaseb McCon

**Affiliations:** West Chester University, West Chester University of PA, Pennsylvania, United States

## Abstract

Losses associated with late life can lead to meaninglessness, loneliness, isolation, and despair. When facing grief and mourning, life review is an effective psychological process that can be healing. Focusing on past strengths and contributions can be helpful when faced with adversity. This qualitative study examined the coping strategies of a diverse group of adults who experienced a significant loss. Utilizing a life review process, we explored how memories of the past could help manage current realities. We identified activities that were healing and fostered meaning, purpose, and ego integrity. Interviews were conducted with 23 men and women over 70. Participants were a part of a nine-year “Life History Project” (2016 – 2025), a part of the WHO and AARP’ Livable Communities Project. All participants (16 women and 7 men) experienced a significant loss during the time of the study. Interviews explored the ways they coped with changing personal and social realities, mourned their losses, and managed their grief. Results of thematic analysis indicated that the primary challenges were 1) personal losses (health and death of loved ones), 2) cultural bereavement (loss of social trust), and 3) stress and concern about the future of the planet (climate anxiety). Effective coping included three interrelated strategies: transcendence (religious/spiritual and natural connections), creative activities (music, painting, writing), and personal and cultural rituals. Transcendence, in particular, represented a shift from egocentricity to a broader awareness of self and the world and resulted in increased peace, acceptance, and coherence of past experiences with present realities.

